# Epidemiology of foot‐and‐mouth disease outbreaks in Thailand from 2011 to 2018

**DOI:** 10.1111/tbed.14754

**Published:** 2022-11-22

**Authors:** Thanicha Chanchaidechachai, Helmut Saatkamp, Mart de Jong, Chaidate Inchaisri, Henk Hogeveen, Sith Premashthira, Noppawan Buamitoup, Rotchana Prakotcheo, Bart H. P. van den Borne

**Affiliations:** ^1^ Business Economics Group Wageningen University Wageningen The Netherlands; ^2^ Quantitative Veterinary Epidemiology Group Wageningen University Wageningen The Netherlands; ^3^ Research Unit of Data Innovation for Livestock Department of Veterinary Medicine Faculty of Veterinary Science Chulalongkorn University Bangkok Thailand; ^4^ Bureau of Disease Control and Veterinary Services Department of Livestock Development Bangkok Thailand

**Keywords:** foot‐and‐mouth disease, risk factors, spatial analysis, temporal analysis, Thailand

## Abstract

Foot‐and‐mouth disease (FMD) is one of the most important animal diseases hindering livestock production in Thailand. In this study, a temporal and spatial analysis at the subdistrict level was performed on FMD outbreak reports in Thailand from 2011 to 2018. Risk factors associated with FMD outbreaks were furthermore investigated using generalized estimating equations. The results showed that the incidence of FMD outbreaks was the highest in 2016 and was affected by season, with a peak in FMD outbreaks occurring in the rainy‐winter season, during October to December. FMD outbreaks were mostly distributed in small clusters within a few subdistricts. Some high‐risk areas with repeated outbreaks were detected in the central regions. Risk factors, including the increase of subdistrict's size of the dairy population, beef population or pig population, the low percentage of forest area, subdistricts in the provinces adjacent to Malaysia, the presence of a livestock market and the occurrence of an FMD outbreak in a neighbouring subdistrict in the previous month significantly increased the odds of having an FMD outbreak. The increase in proximity to the nearest subdistrict with an FMD outbreak in the previous month decreased the odds of having FMD outbreaks. This study helped to identify high‐risk areas and periods of FMD outbreaks in Thailand. Together with the identified risk factors, its results can be used to optimize the FMD control programme in Thailand and in other countries having a similar livestock industry and FMD situation.

## 1 INTRODUCTION

Driven by domestic consumption and exports of animal products, livestock production in Thailand has been gradually growing since the late 1970s when farming systems in Thailand started to shift from extensive farming to intensive farming with an accompanying increase in farm size (Delgado et al., 2008). However, the growth of the country's livestock industry is hindered by multiple constraints, including infectious animal diseases, of which foot‐and‐mouth disease (FMD) is the most important in terms of economic impact (Perry et al., 1999).

Foot‐and‐mouth disease virus (FMDV) belongs to the *Aphthovirus* (family Picornaviridae) and can infect cloven‐hooved animals. FMDV consists of seven immunologically distinct serotypes, O, A, C, Asia 1, SAT 1, SAT 2 and SAT 3 (Davies, 2002). Mainland South‐East Asia (SEA), which encompasses Peninsular Malaysia, Myanmar, Vietnam, Laos, Cambodia and Thailand, is endemic for serotypes O, A and Asia 1 (Knowles et al., 2012). Even with an annual vaccination programme in place, 928 FMD outbreaks, distributed all over Thailand, have been reported to the ASEAN Regional Animal Health Information System from 2007 to 2017 (Blacksell et al., 2019). The risk of FMD outbreaks in Thailand has increased because of the increase of livestock trading in the SEA region due to a rise in livestock demand from China (Smith et al., 2015).

Area‐specific knowledge of the epidemiology of FMD is fundamental to improving preventive measures and control strategies, as insight into the occurrence of FMD can help authorities allocate resources to areas at greater risk. Studies in a variety of FMD endemic areas showed multifaceted risk factors related to FMD, including the type of animal species (Nyaguthii et al., 2019; Yano et al., 2018), production system (Megersa et al., 2009), presence of a livestock market (Jemberu et al., 2016), livestock traders (Souriya et al., 2020), adjacency to a national park (Allepuz et al., 2015) and a seasonal effect (Guerrini et al., 2019). Studies in SEA highlighted the importance of transboundary animal movement as the major risk of FMD transmission (Blacksell et al., 2019; Madin, 2011).

Most of the studies on FMD patterns and risk factors in Thailand were conducted at the regional level. For example, Arjkumpa et al. (2020) studied spatiotemporal clusters of FMD outbreaks in the northern part of Thailand. A study focusing on Northern Thailand dairy farms showed that farms located near communal grazing areas or slaughterhouses and imported cattle without quarantining animals were at greater risk for FMD (Sansamur et al., 2020). Another study from Sangrat et al. (2020) used experts’ opinions to evaluate the weight of spatial risk factors associated with the occurrence of FMD in Thailand and used it to create a risk map. The authors found that the risk of FMD occurrences increased in areas close to a region with a previous outbreak, livestock markets, slaughterhouses, boundary lines and areas with a high density of beef cattle, pigs, dairy cattle, buffaloes, humans and roads (Sangrat et al., 2020). However, a study investigating the patterns and risk factors of FMD at the country level based on actual outbreak data is lacking.

In this study, the spatial and temporal patterns of FMD outbreaks in Thailand and the associated risk factors were identified based on subdistrict level data of FMD outbreaks reports from 2011 to 2018.

## 2 MATERIALS AND METHODS

### 2.1 Study area

Thailand is a country located in the middle of Mainland SEA with an area of 513,120 km^2^. The land border is adjacent to Myanmar, Laos, Cambodia and Malaysia. The administration structure consists of 77 provinces and subdivides into the local administration of districts (*n* = 878) and subdistricts (*n* = 7425). The topographical features include a central plain, the upland plateau in the north‐eastern region and the high mountains, which cover Northern Thailand and extend along the Myanmar border to the Malaysia peninsula. The elevation varies from sea level up to 2562 m in the mountainous areas. The climate is divided into three seasons. The rainy season stretches from mid‐May to mid‐October. The winter season extends from mid‐October to mid‐February, during which most parts of the country experience dry weather with mild temperatures. Finally, the summer season stretches from mid‐February to mid‐May (Thai Meteorological Department, 2015).

Livestock species typically affected by FMD are cattle, buffalo, small ruminants and pigs. The geographical distribution of livestock in Thailand is diversified. The majority of pig farms and dairy farms are located in the central, western and eastern regions. Beef cattle and buffalo farms are concentrated in the north‐eastern region, whereas most small ruminant farms are located in the southern region of Thailand (Department of Livestock Development, 2020).

The main control measure of FMD in Thailand is routine vaccination. It is supported by the Thai government by distributing free of charge trivalent FMD vaccines (O, A and Asia1 serotypes) for dairy cattle and a bivalent FMD vaccine (O and A serotypes) for beef cattle, buffaloes and small ruminants. FMD vaccines are produced by the Bureau of Veterinary Biologics Pak Chong facility in Thailand, distributed to local veterinary offices and the dairy cooperative network, and then to individual farmers, local veterinary officers and private farm veterinarians who vaccinate the animals. Vaccination of ruminants is compulsory. Dairy cattle are vaccinated three times yearly, whereas small ruminants, beef cattle and buffalo are vaccinated twice a year (Arjkumpa, Sansamur, et al., 2020; Arjkumpa, Yano, et al., 2020). The government also produces a trivalent FMD vaccine (O, A and Asia 1 serotypes) for pigs. It is commercially available and sold at a below‐market price due to government subsidy (Yano et al., 2018) but pig farmers can also use other commercially available FMD vaccines (unpublished data from FMD project [PRP 5905021280]). The vaccination programme in pigs is voluntary and depends on the farmers’ own willingness to participate.

The Thai FMD surveillance programme consists of both active and passive surveillance components. Active surveillance includes routine visits to cattle farms by local veterinary officers and monitoring the FMD status of imported cattle. Passive surveillance means that farmers are expected to report FMD suspected cases upon notification to local veterinary officers (Arjkumpa, Yano, et al., 2020). When FMD outbreaks are reported, control measures are immediately implemented in order to stop disease transmission, including the quarantining of suspected premises, outbreak areas announcements, animal movement control and ring vaccination (Yano et al., 2018).

### 2.2 Data collection

Subdistrict level FMD outbreak data between 2011 and 2018 were acquired from the Department of Livestock Development. Recorded data consisted of the name of the subdistrict having the outbreak, the starting date of the outbreak, the population size and the livestock species involved. The outbreaks were clinically diagnosed by local veterinary officers, with at least one animal in the area showing the typical signs of FMD and confirmed by laboratory tests using ELISA, virus isolation, PCR or a combination of those, at the Regional Reference Laboratory for FMD in SEA.

Data on putative subdistrict level risk factors associated with FMD outbreaks were collected from various sources. The subdistrict level data of livestock population, consisting of species and population size, were obtained from livestock census data. Due to a lack of completeness, census data were only available in 2013, 2015 and 2018. Therefore, the 2013 census for the livestock population was used for 2011 to 2013. The 2015 census for livestock population was used for 2014 to 2016, and the 2018 census for livestock population was used for 2017 to 2018. Monthly rainfall in Thailand data between 2011 and 2018 was obtained from the National Oceanic and Atmospheric Administration. Data on elevation, forest area and adjacency to neighbour countries were extracted from the Thailand raster map using QGIS 3.4 (QGIS Development Team, 2020). Locations of livestock markets and slaughterhouses were obtained from the Department of Livestock Development database (Department of Livestock Development (DLD), 2019).

### 2.3 Statistical analysis

#### 2.3.1 Temporal analysis

Time series of monthly FMD outbreak reports were created. If a new outbreak in the same subdistrict was reported within 30 days, we counted them as one outbreak, and only the date of the first outbreak report was analysed. Seasonal and trend decomposition with locally estimated scatterplot smoothing (STL) was applied to analyse seasonal effects and trends. STL is a method to decompose time series into three additive components (trend, seasonality and remainder) using locally estimated scatterplot smoothing, which is the process of smoothing a regression curve to data points. The components can be written as follows:

$$Y_{v}=\;T_{v}+\;S_{v}+\;R_{v}$$

where *Y* is the series value; *T* is the trend component; *S* is the seasonal component; *R* is the remainder at time *v*. Seasonal and trend decomposition with locally estimated scatterplot smoothing started with the outer loop by estimating the trend component and assigning a robustness weight to each data point. The weight depended on the size of the remainders, which reduced the effect of outliers. The trend was subtracted from the raw data to detrend time series. Then, the detrended series was passed to the inner loop where the seasonal component was estimated using locally estimated scatterplot smoothing cycle subseries (for the yearly seasonal, there were 12 cycle subseries, i.e. January to December). The outer loop was iteratively updated with the new trend components estimated by subtracting the estimated seasonal component from the raw data. The new detrended series was passed to the inner loop again to update the seasonal component. The process continued until the setting number of cycles was reached. The variation that was not explained by the seasonal and trend components was considered the remainder (Cleveland et al., 1990). The remainders were checked for autocorrelation to ensure that no trend and the seasonal effect were left in the remainders. The strength of the trend (*F*_T_) and seasonal (*F*_S_) component on time series can be measured by

$$F_{T}=\;1-\;\frac{\mathrm{Var}\left(R_{v}\right)}{\mathrm{Var}\left(T_{v}+R_{v}\right)}$$

$$F_{S}=\;1-\;\frac{\mathrm{Var}\left(R_{v}\right)}{\mathrm{Var}\left(S_{v}+R_{v}\right)}$$

The measure of the strength of each component has a value between 0 and 1. A value close to 1 shows a strong effect (Hyndman & Athanasopoulus, 2018), whereas a value close to 0 indicates no effect from the component. The STL method was applied using the stl package in R program version 3.6 (R Core Team, 2022).

#### 2.3.2 Spatial analysis

The spatial distribution of the risk of subdistricts experiencing an FMD outbreak was described by calculating standardized morbidity ratios (SMRs) at the subdistrict level for each year. SMR is the ratio of observed number of cases relative to the expected number of cases in each subdistrict, where a case was defined as an animal with clinical signs of FMD. The expected cases for each subdistrict were calculated by multiplying the incidence rate from the entire population, which equals the total number of cases divided by the total population at risk, with the size of the population at risk in each subdistrict. If SMR > 1, the risk for that subdistrict is higher than the national risk. Therefore, SMR can be interpreted as a relative risk (Waller & Gotway, 2004). Estimated SMRs for areas with a low animal population may be imprecise because of variance instability. An empirical Bayes estimator with a Poisson–Gamma model was therefore applied to improve SMR estimates (Clayton & Kaldor, 1987). The number of years that subdistricts had SMRs > 1 was depicted in a choropleth map. The higher the number of years that subdistricts had SMRs > 1, and the more likely these subdistricts were hot spot areas of FMD outbreaks. The heterogeneity of SMRs was assessed using a Chi‐squared test. The global Moran's *I* index was used to quantify the spatial autocorrelation of SMRs. The null hypothesis is that SMRs are randomly distributed among the areas. A positive Moran's *I* index implies a clustering of high or low SMRs in the same neighbourhood area. A negative Moran's *I* index implies the dispersion of high or low SMRs (Bivand et al., 2013). Moran's *I* index can be calculated by

$$I=\frac{N\;\sum_{i}\sum_{j}w_{ij}\left(\mathrm{SM}R_{i}-\bar{\mathrm{SMR}}\right)\left(\mathrm{SM}R_{j}-\bar{\mathrm{SMR}}\right)}{\left(\sum_{i}\sum_{j}w_{ij})(\mathrm{SM}R_{i}-\bar{\mathrm{SMR}}\right)}$$

where *N* is the number of spatial units indexed by *i* and *j*; SMR_*i*_ is the SMR for subdistrict *i*; SMR_*j*_ is the SMR for subdistrict *j*; $w_{ij}$ is the inverse of the distance between the centroid of subdistrict *i* and the centroid of subdistrict *j* (Gómez‐Rubio et al., 2005). The spatial analyses were conducted for all years combined and each year separately and were performed in R program version 3.6 (R Core Team, 2022) using the DCluster Package.

A discrete Poisson scan statistic was performed using SaTScan software, version 9.6 (Kulldorff, 2020) to detect the location of spatial clusters of FMD cases consisting of the number of animals with FMD. The centroid of subdistricts was assumed to represent the outbreak location. The subdistrict name, the number of animals with FMD, the animal population in each subdistrict and the geographical coordinates of subdistrict centroids were provided as input files. The expected numbers of FMD cases were calculated based on the animal population in each subdistrict using a Poisson distribution. Circular windows of varying sizes were then scanned over the space to find clustering of FMD cases in the same area. Clusters of FMD were defined where the risk inside the window exceeded the risk outside the window. The likelihood ratio test was used to determine the most likely cluster. The *p‐*value is based on Monte Carlo hypothesis testing by comparing the maximum likelihood of actual data with randomly generated data (Kulldorff, 2018). The maximum spatial cluster size was set at 50% of the population at risk. The minimum spatial cluster size was set at more than 50 FMD cases to avoid cluster detection due to a small population at risk. The Gini index was used to select the reported clusters (Han et al., 2016), and clusters were not allowed to overlap. Only clusters with a significance level <0.05 were reported. The scan statistics analysis was run for each year to remove the effect of long‐term population structure changes. Subdistricts that had centroids located within significant yearly spatial clusters were reported in the maps.

#### 2.3.3 Risk factor analysis

Generalized estimating equation logistic regression models, to accommodate for the autocorrelation of monthly reported data within subdistricts, were used to identify risk factors associated with FMD outbreaks. The dependent variable is the monthly occurrence of an FMD outbreak in each subdistrict. Putative subdistrict level risk factors included: (1) the species‐specific population size of livestock in each subdistrict, that is dairy cattle, beef cattle, buffalo, small ruminants and pigs; (2) monthly rainfall; (3) elevation; (4) international border contact, defined as being a subdistrict located in a border province; (5) the presence of a slaughterhouse; (6) the presence of a livestock market; (7) a historical FMD outbreak in a neighbouring subdistrict in the previous month (a neighbouring subdistrict was defined as a subdistrict sharing adjacent borders to the subdistrict under scrutiny); (8) proximity to the nearest subdistrict with an FMD outbreak in the previous month; (9) the percentage of forest area, which is calculated by dividing the forest area by the total subdistrict area.

Year was included as a categorical variable in the model to correct the yearly trend in FMD outbreaks. Month was included as a sine–cosine function of the numerical month to present the seasonal fluctuation (Stolwijk et al., 1999). The basic statistical model can be written as follows:

$$\ln\left(\frac{p}{1-p}\right)=\;\alpha +\;\beta_{1}\times \sin\left(2\pi \times \frac{m}{12}\right)+\;\beta_{2}\times \cos\left(2\pi \times \frac{m}{12}\right)$$

where *p* is the probability of having an FMD outbreak in a particular month; α is the intercept; *m* is the numerical month (1–12 for January–December). Due to the non‐linear relationship between some independent variables and the log‐odds of having an FMD outbreak, the continuous variables of the species‐specific livestock population size and the proximity to the nearest subdistrict with FMD outbreak variables were log_10_‐transformed. The percentages of the forest area and monthly rainfall were categorized based on three quantile densities. The exchangeable correlation structure was selected based on the lowest QIC of null models (Cui & Qian, 2007). Univariable analysis was performed for all putative risk factors, and variables with a *p*‐value <0.15 for the Type 3 test were eligible for the multivariable analysis. Correlation between the selected variables was checked. If the correlation coefficient was >0.5, one of the correlated variables was selected. The correlation analysis showed a high correlation between elevation and international border and between rainfall and month of the year, which is not a surprise given that most mountainous ranges of Thailand stretch into neighbouring countries and the seasonality of rainfall. As several studies showed that international border is an important risk factor of FMD outbreak, we kept the international border and month, whereas elevation and rainfall were excluded as the possible explanatory variables (Allepuz et al., 2015; Hamoonga et al., 2014; Picado et al., 2011). Subsequently, a backward selection process was applied to identify all variables significantly (*p*‐value <0.05) associated with FMD outbreaks. The change of coefficient after dropping variables was checked to confirm that the dropping variables were not confounders. From the final statistical model, the predicted probability for having an FMD outbreak in the subdistrict for each month was calculated as follows:

$$\mathrm{probability}\;\mathrm{of}\;\mathrm{event}\;=\;\frac{1}{1+\;e^{-\left(\beta_{0}+\beta_{1}X_{1}+\;\beta_{2}X_{2}+\cdots \right)}}$$

The predicted probability was compared with the actual FMD outbreak report to visually evaluate the model fit when geographically being displayed in a map. We measured the correspondence between the predicted probabilities and outbreak incidences using Pearson correlation.

## 3 RESULTS

### 3.1 Temporal analysis

**FIGURE 1:**
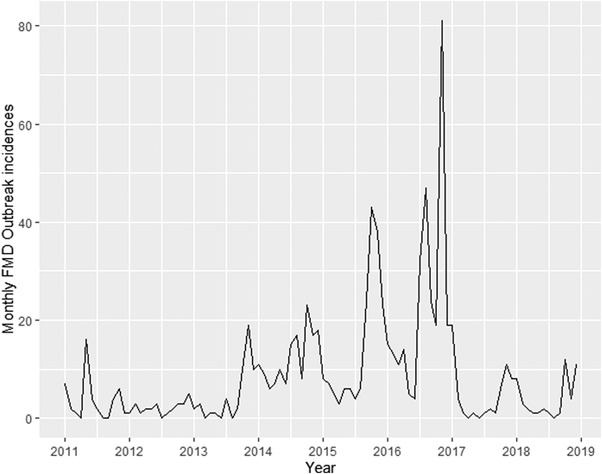
Monthly reported incidence of foot‐and‐mouth disease outbreaks in Thailand from 2011 to 2018

**FIGURE 2:**
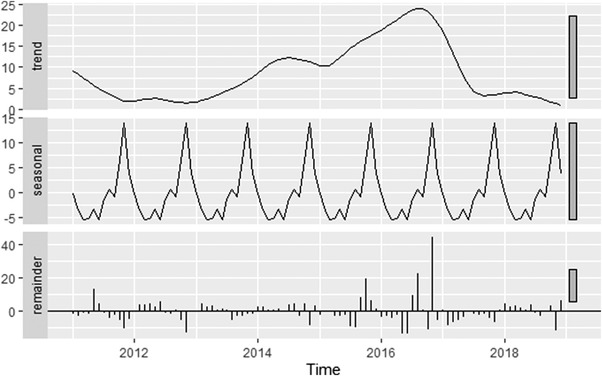
The decomposition of the monthly incidence of foot‐and‐mouth disease outbreaks in Thailand from 2011 to 2018 into a trend, seasonal and remainder component. The grey bars on the right side are scale bars of equal measurement, indicating the relative scale of each component.

From 2011 to 2018, 826 FMD outbreaks were reported. The monthly incidence of FMD outbreak reports is plotted in Figure 1. The median monthly incidence of FMD outbreak reports was 4 (min = 1, max = 81, mean = 9). The highest yearly incidence of FMD outbreak reports was in 2016 (*n* = 284), and the lowest was in 2011 and 2012 (*n* = 43). The decomposition plot is shown in Figure 2. As can be observed from the trend component, the incidence of FMD outbreaks increased from 2011 onwards and reached a peak in 2016 before decreasing. The seasonal component showed that the incidence of FMD outbreaks was low from February to June before increasing and reaching the peak at October–December. The measure of strength for the trend and seasonal components were 0.51 and 0.36, respectively, indicating that the effect of the trend component on the monthly incidence of FMD outbreak reports was more substantial than the effect of the seasonal component.

### 3.2 Spatial analysis

**FIGURE 3:**
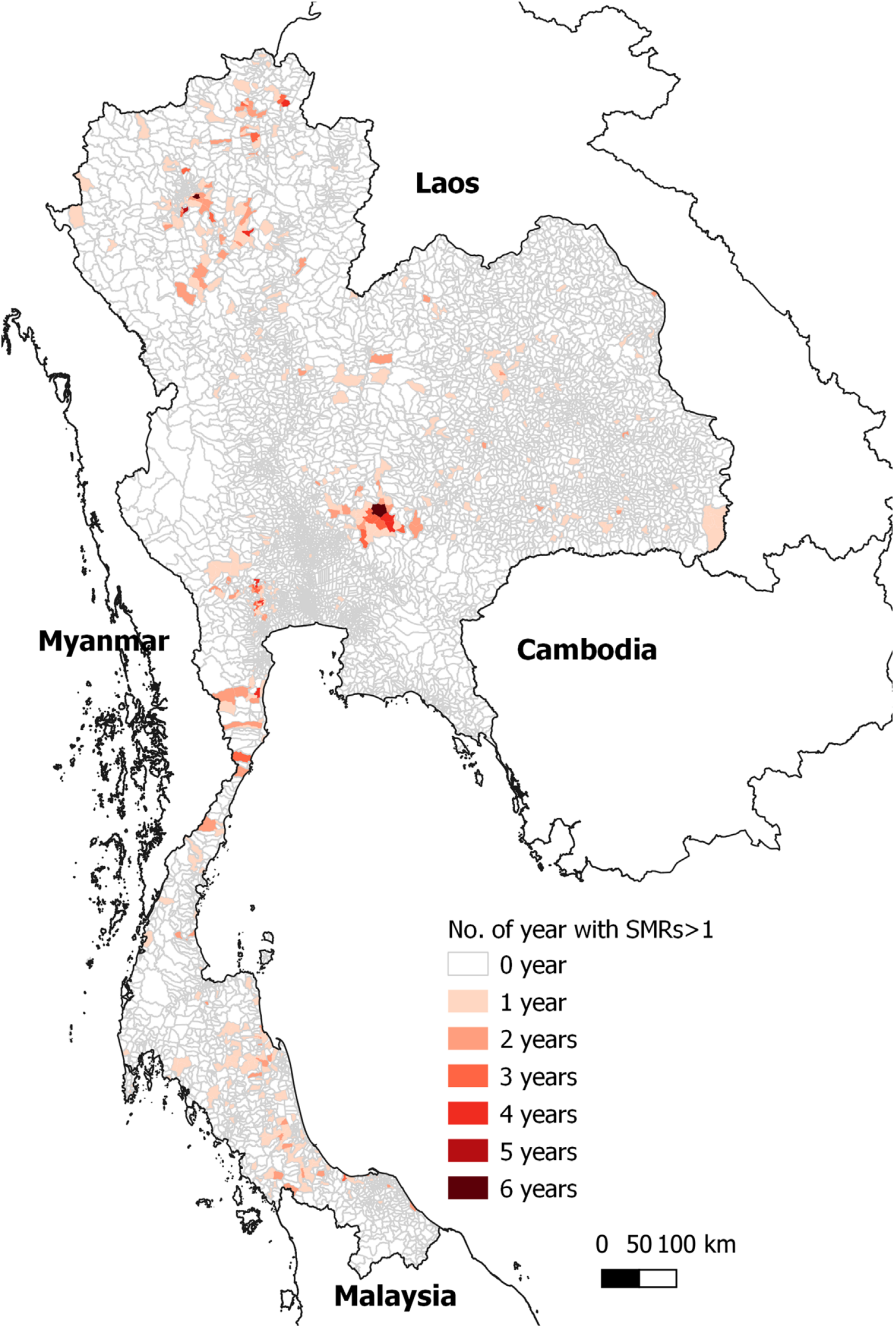
The number of years that subdistricts in Thailand had standardized morbidity ratios (SMRs) of foot‐and‐mouth disease outbreaks higher than one from 2011 to 2018.

**FIGURE 4:**
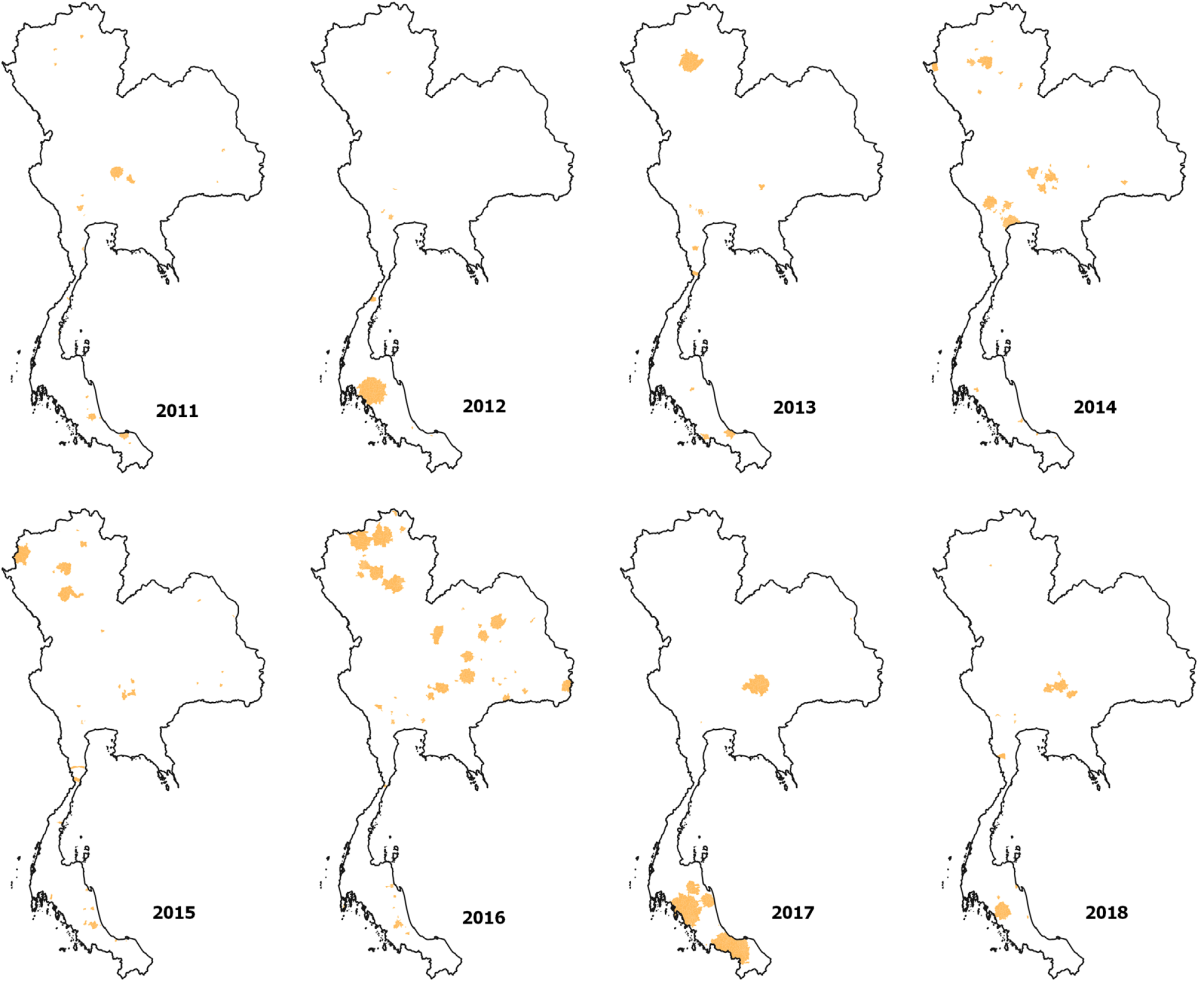
Locations of significant spatial clusters of foot‐and‐mouth disease outbreaks in Thailand from 2011 to 2018

From the 7425 subdistricts in Thailand, 564 subdistricts reported at least one FMD outbreak. The subdistrict with the highest frequency of FMD outbreak reports was Lamphaya Klang subdistrict in Saraburi province, which is located in Central Thailand, with 12 FMD outbreaks reported from 2011 to 2018. The geographical distribution of subdistricts having SMRs > 1 is depicted on a choropleth map in Figure 3. The Chi‐squared tests showed that the number of observed cases was different from the expected risk in some subdistricts (Table 1). Significant positive Moran's *I* indices were observed in 2011, 2012, 2013, 2016 and 2017 (Table 1), indicating clustering of high or low SMRs in the same neighbourhood area, although Moran's *I* indices values were low. The spatial clusters of similar SMRs in the same areas are depicted in Figure 4. Statistically significant spatial clusters were detected in 2011–2018. The reported clusters tended to be small clusters within a few subdistricts rather than being large clusters. The clusters were mostly located in the central, southern and northern regions, except in 2016 when clusters were much more dispersed. The annual number of significant clusters varied from 8 clusters (year 2012) to 51 clusters (year 2016).

**TABLE 1:** Presence of global spatial patterns of foot‐and‐mouth disease outbreaks in Thailand in 2011 until 2018 based on the Chi‐squared test for the difference between expected risk and observed standardized morbidity ratios (SMRs) and the global Moran's *I* index

Year	Chi‐squared test	Moran's *I* index	Moran's *I p* value
2011	0.001	0.004	0.034
2012	0.001	0.03	0.001
2013	0.001	0.005	0.016
2014	0.001	0	0.291
2015	0.001	0	0.134
2016	0.001	0.037	0.001
2017	0.001	0.007	0.015
2018	0.001	0.002	0.098

### 3.3 Risk factor analysis

Descriptive statistics of putative risk factors are shown in Tables 2 and 3. The distribution of the subdistrict had a high disparity for some risk factors. For example, there were relatively few subdistricts having a livestock market and a neighbouring subdistrict that experienced an FMD outbreak in the previous month.

**TABLE 2:** Mean and standard error of continuous risk factors for foot‐and‐mouth disease (FMD) outbreak at the subdistrict level

Variable	Mean ± standard error	
	Subdistrict with FMD outbreaks	Subdistrict without FMD outbreaks
Log_10_ (dairy population)	1.49 ± 1.61	0.26 ± 0.71
Log_10_ (beef population)	2.75 ± 0.64	2.49 ± 0.78
Log_10_ (buffalo population)	1.10 ± 0.95	1.34 ± 1.05
Log_10_ (small ruminant Population)	1.22 ± 1.18	0.70 ± 1.00
Log_10_ (pig population)	2.61 ± 1.13	2.16 ± 1.11
Log_10_ (proximity to the nearest subdistrict with the outbreak in the previous month)	2.05 ± 0.06	2.29 ± 1.01

**TABLE 3:** Descriptive analysis of putative categorical risk factors for foot‐and‐mouth disease (FMD) outbreak at subdistrict‐month level

Variable	Categories	No. of subdistrict‐month per categories (total *n* = 668,724)	FMD outbreak incidence per subdistrict‐month
Monthly rain fall	<50 mm	225,060	1.03 × 10^−3^
	≥50 and <180 mm	219,724	1.46 × 10^−3^
	≥180 mm	223,940	1.21 × 10^−3^
Elevation	<36 m	200,856	1.23 × 10^−3^
	≥36 and <165 m	231,156	0.90 × 10^−3^
	≥165 m	236,712	1.55 × 10^−3^
Percentage of forest area	0%	552,576	1.18 × 10^−3^
	>0% and ≤33%	63,240	1.53 × 10^−3^
	>33% and ≤66%	36,552	1.67 × 10^−3^
	>66%	16,356	0.79 × 10^−3^
International border contact	No contact	412,608	1.15 × 10^−3^
	Cambodia	67,260	0.46 × 10^−3^
	Malaysia	28,428	1.86 × 10^−3^
	Laos	91,104	0.97 × 10^−3^
	Myanmar	69,324	2.57 × 10^−3^
Presence of slaughter house	No	543,996	1.10 × 10^−3^
	Yes	124,728	1.83 × 10^−3^
Presence of livestock market	No	658,416	1.22 × 10^−3^
	Yes	10,308	2.33 × 10^−3^
FMD outbreaks in neighbouring subdistrict in the previous month	No	666,331	1.03 × 10^−3^
	Yes	2393	57.25 × 10^−3^

After correcting for seasonal and yearly trends, the final statistical model included eight statistically significant risk factors (Table 4). A 10‐fold increase in the population size of dairy cattle, beef cattle or pigs increased the odds of having FMD outbreaks 2.06, 1.43 or 1.22 times, respectively. The proximity to the nearest subdistrict with an FMD outbreak in the previous month was inversely related to the odds of having an FMD outbreak. A 10‐fold closer proximity increased the odds of having FMD outbreaks three (=1/0.33) times. The areas with a low percentage of forest area had 1.29 times higher odds of having FMD outbreak than subdistricts without forests. Subdistricts located in the provinces neighbouring Malaysia had 2.61 times higher odds of FMD outbreak than the subdistricts located in provinces without international borders. Subdistricts having a neighbouring subdistrict with FMD outbreak in the previous month had 4.41 times higher odds of having an FMD outbreak compared to subdistricts not experiencing an FMD outbreak in any of their subdistricts in the previous month.

**TABLE 4:** The final risk factor model for foot‐and‐mouth disease (FMD) outbreak at subdistrict level with coefficients, standard error, odds ratio and statistical significance level

Variable	Coefficients	Standard error	Odds ratio (95%CI)	*p*‐value
Log_10_ dairy population	0.72	0.04	2.06 (1.90–2.22)	<0.001
Log_10_ beef population	0.36	0.08	1.43 (1.22–1.67)	<0.001
Log_10_ pig population	0.20	0.04	1.22 (1.12–1.32)	<0.001
Log_10_ proximity to the nearest subdistrict with an outbreak in the previous month	−1.09	0.06	0.33 (0.30–0.38)	<0.001
Percentage of forest area				
0% (none)	Ref.			
>0% and ≤33% (low)	0.25	0.13	1.29 (1.01–1.65)	0.04
>33% and ≤66% (medium)	0.28	0.16	1.32 (0.96–1.81)	0.08
>66 % (high)	0.008	0.27	1.01 (0.60–1.71)	0.98
International border contact				
No contact	Ref.			
Cambodia	−0.43	0.20	0.65 (0.44–0.97)	0.03
Laos	0.29	0.15	1.33 (0.99–1.76)	0.05
Malaysia	0.96	0.18	2.61 (1.84–3.71)	<0.001
Myanmar	−0.17	0.11	0.84 (0.68–1.03)	0.11
Presence of livestock market				
No	Ref.			
Yes	0.55	0.30	1.74 (0.96–3.16)	0.068
FMD outbreaks in neighbouring subdistricts in the previous month				
No	Ref.			
Yes	1.48	0.13	4.41 (3.42–5.68)	<0.001
Year				
2011	Ref.			
2012	−0.43	0.25	0.65 (0.40–1.05)	0.08
2013	−0.06	0.21	0.94 (0.63–1.41)	0.77
2014	0.96	0.17	2.62 (1.87–3.67)	<0.001
2015	1.09	0.17	2.99 (2.15–4.14)	<0.001
2016	1.48	0.16	4.37 (3.19–5.99)	<0.001
2017	0.24	0.21	1.27 (0.85–1.91)	0.24
2018	0.07	0.22	1.07 (0.69–1.66)	0.75
Month				
$\sin(2\pi \times \frac{m}{12})$	−0.38	0.05	–	<0.001
$\cos(2\pi \times \frac{m}{12})$	0.44	0.05	–	<0.001

**FIGURE 5:**
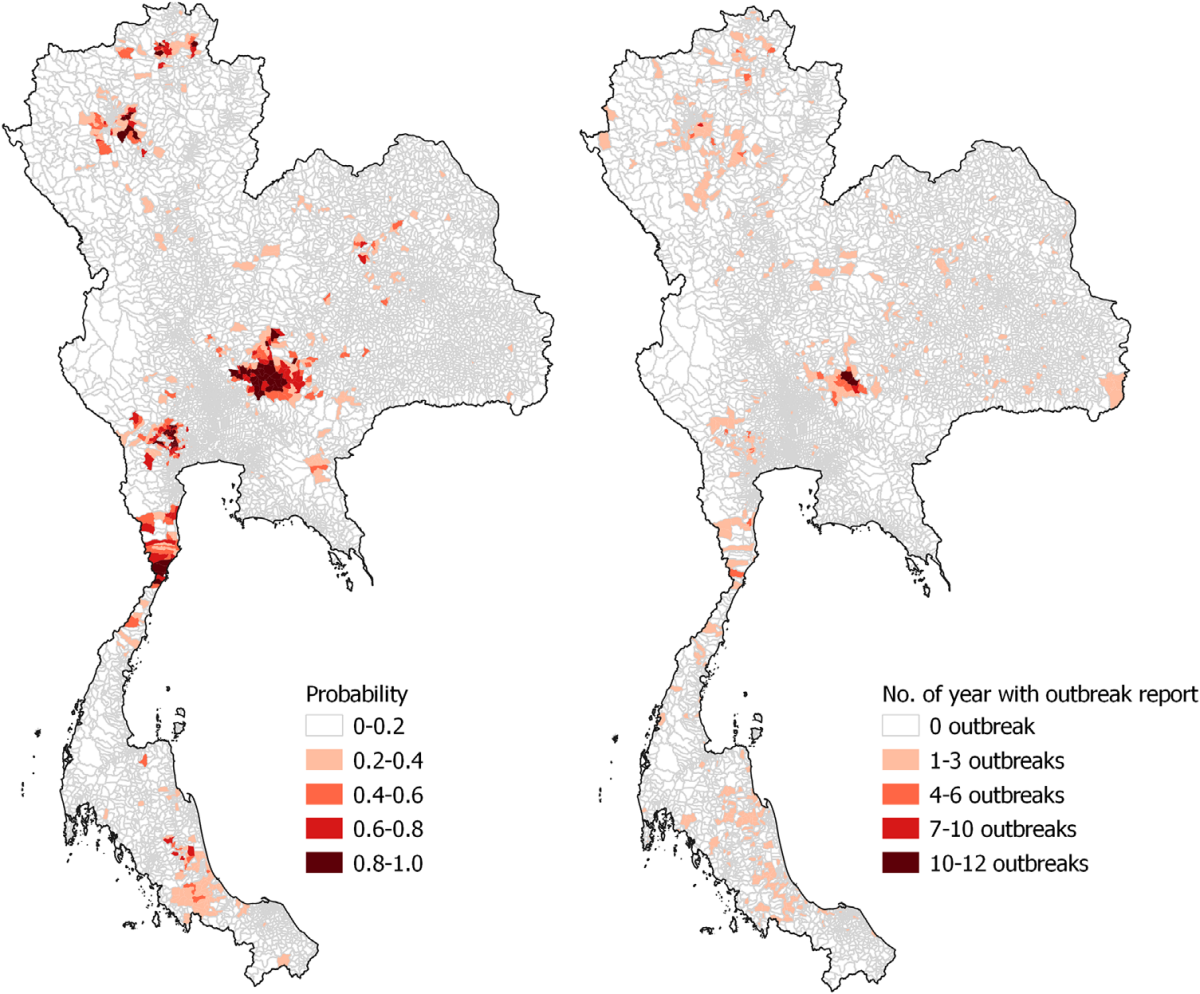
Geographical distribution of the predicted probability of foot‐and‐mouth disease outbreak resulting from the final statistical model (left) and the frequency of subdistrict‐year level foot‐and‐mouth disease outbreak reports in Thailand from 2011 to 2018 (right).

The predicted probability of having an FMD outbreak at the subdistrict level was compared with the actual FMD outbreak report (Figure 5). The high probability areas were mostly clustered in the central region and distributed as small patches in the north and southern part of Thailand. The predicted probabilities corresponded with the observed incidences with a Pearson correlation coefficient of 0.55.

## 4 DISCUSSION

This study used reported outbreak data to describe the temporo‐spatial distribution of FMD outbreaks in Thailand and to identify associated risk factors and is one of the few studies conducted in FMD endemic areas attempting to control the disease using vaccination. The number of FMD outbreaks was higher in 2016 than in other years as indicated by the highest trend component in 2016. There is seasonal variation in the occurrence of FMD outbreaks, with the number of FMD outbreaks being the lowest in the summer season (March to May) and the highest in the rainy to winter season (October to November). The seasonal trend contradicts the results from previous studies in Africa, where the peak of FMD incidence occurred during the hot and dry season because of an increase in animal movement to forage food and water sources (Ayebazibwe et al., 2010). However, livestock production in Thailand is more intensive and industrial, with livestock staying inside the farm areas without having a need to move around to forage for food, except for small beef cattle and buffalo farms. Another possible explanation is the effect of temperature and relative humidity on FMDV environmental survival. FMDV is expected to survive longer at low temperatures and higher relative humidity (Mielke & Garabed, 2020). This reason can explain a lower number of FMD outbreaks in dry summer and a peak in the rainy to the winter season when the temperature drops, and the relative humidity remains high. Another possible explanation is a potential delay of vaccine delivery and vaccination practices in the rainy season, resulting in lower vaccination coverage, especially for smallholder farms in rural areas.

The results from the spatial analysis clearly showed that in some subdistricts, the incidence of FMD was higher than in other subdistricts. Certain regions are more vulnerable to the repetitive occurrence of FMD outbreaks, for which the risk factor analysis has provided some useful insights. The majority of FMD outbreaks tended to occur as small clusters within a few subdistricts rather than extending to a larger area. The limited size of the FMD outbreaks might be explained by the immunity level in the population resulting from vaccination and the natural immunity from previous outbreaks (Pomeroy et al., 2015). An outbreak is self‐limited when the population of susceptible animals in the area is depleted to the point that herd immunity is reached (Estrada et al., 2008).

The occurrence of outbreaks, despite a vaccination programme being in place, implied a lack of herd immunity from the vaccination programme. Several factors could be related to the vaccination inefficiency, such as a poor duration of vaccine‐induced immunity (Knight‐Jones et al., 2015), low matching of the vaccine with field strains (Mahapatra & Parida, 2018) and low vaccination coverage (Wataradee et al., 2021), especially in pig farms where the FMD vaccination programme is not mandatory. The variation in FMDV strains might explain the high FMD incidence in 2016. The sequences of FMDV collected from FMD outbreaks in 2016 were classified as O/SEA/Mya‐98 lineage, which differed from the sublineages Mya‐98a that caused the FMD outbreaks in Thailand in 2009 (unpublished data from FMD project, Agricultural Research Development Agency, Thailand Research Fund (PRP 5905021280)). Moreover, a new strain–O/ME‐SA/Ind‐2001d was reported in 11 provinces of Thailand in 2016 (WAOH, 2017). The co‐infection of FMDVs and intra‐host recombination could increase the genetic diversity of FMDV (Aiewsakun et al., 2020). Nevertheless, it is uncertain whether the outbreaks in Thailand result from ongoing transmission or from reintroductions from other locations. Further studies should be conducted to investigate why FMD outbreaks still occur in Thailand, even with vaccination.

The odds of FMD outbreak occurrence increased with the increasing size of the dairy cattle, beef cattle or pig populations in the subdistrict. Similarly, the size of the forest area was also a significant risk factor, which is a likely surrogate for farm density. These results were expected as a higher number of susceptible hosts may increase the chances of virus circulation. The varying odds amongst the different livestock species might be explained by the fact that different species vary in susceptibility and transmissibility of FMDV. Cattle are considered to be the most susceptible species, whereas pigs are the most infectious species (Bravo de Rueda et al., 2015). The occurrence of an FMD outbreak in a neighbouring subdistrict in the previous month and the proximity to the nearest outbreak were also significant factors. Both supported the possibility of an FMD outbreak through local transmission.

Previous studies suggested that transboundary transmission is a significant risk factor for FMD outbreaks in the SEA region (Blacksell et al., 2019; Smith et al., 2015). In our study, we found that subdistricts in provinces having international borders with Malaysia had a higher incidence of FMD outbreaks compared to subdistricts located in provinces not having an international border. Thailand is a transit country for large ruminants from Myanmar to higher value markets in Malaysia via the southern borders (Smith et al., 2015; Wongsathapornchai et al., 2008) and China via the northern provinces and subsequently the Laos–Vietnam route to China (Angkuraseranee et al., 2019; Bunmee et al., 2018). These international trade routes pose a risk for transboundary transmission. Despite the lower odds of FMD outbreak occurrence in the subdistricts of provinces bordering Myanmar and Cambodia, it is not possible to exclude the possibility of transboundary transmission between Thailand and these two countries. Due to an extensive amount of shared borders, a large number of ruminants are unofficially moved cross‐border without taking appropriate biosecurity measures, such as quarantining or testing, leading to an increased risk of FMD transmission (Blacksell et al., 2019). The imported animals are typically transferred to animal markets or holding facilities for fattening in other areas (Smith et al., 2015). The studies of the link between animal movement networks and the occurrence of FMD in Thailand should be further conducted.

The results of the risk factor analysis were in accordance with a previous study from Thailand, based on expert opinion, suggesting that the risk of FMD occurrences would increase in areas located close to previous outbreaks, livestock markets, slaughterhouses, boundary lines and areas with a high density of beef cattle, pigs and dairy cattle (Sangrat et al., 2020). The current study supports these findings and quantifies the strength of the associations.

From the probability map, the majority of areas correctly predicted FMD outbreak occurrences, indicating a generally good fit of the model. However, we noted that some areas experienced FMD outbreaks, whereas they had a zero probability of outbreaks, indicating that some underlying factors were not identified in our analysis.

Some potential limitations of this study should be considered. First, the FMD outbreak data in this study are based on the national reporting system which is likely to be affected by reporting bias as shown by several studies that the outbreak reporting underestimated the true prevalence (Siengsanan‐Lamont & Blacksell, 2021; van Andel et al., 2020). In the areas that are susceptible to FMD introduction, such as international border areas and areas with a high number of farms, the local authorities might be more alert and conduct more active surveillance. Therefore, the outbreaks in these areas are more prone to be reported than other areas. The passive surveillance, which depends on farmers reporting FMD cases, might cause under‐reporting due to the concern over animal movement restrictions (Sangrat et al., 2020). Especially for pig farms, which are much more industrialized than ruminant farms, the economic impact from animal movement restriction could be larger. Second, a case definition based on clinical diagnosis, rather than on laboratory testing, might further decrease the sensitivity of a report, especially if the infection happened in small ruminants, in which clinical signs are mild and inapparent (Bravo de Rueda et al., 2015). Third, we did not include animal density and the number of animal movements as potential risk factors in the analysis due to data availability. Still, these factors could be important and should be included in further studies. Fourth, the point geographical coordinates of each outbreak location were unknown. Therefore, the centroid of the subdistrict was taken to approximate the location of the FMD outbreak. The location might not represent the actual situation if the subdistrict is very large or if the outbreak involved multiple subdistricts. Despite those limitations, the available data are validated by reliable sources such as the Department of Livestock Development. The results are therefore expected to represent the actual outbreak situation in Thailand.

The results from this study can be used to improve the FMD control programme in Thailand. This knowledge can help authorities to attribute resources and manage the control programme more effectively by focusing on the areas and periods that are at greater risk of having FMD outbreaks resulting from ongoing, potentially undetected, FMDV transmission. From the results, it is suggested that vaccination should be optimized in areas with a high livestock population, especially if they concern cattle and pigs. Similar to ruminants, the vaccination programme in pigs should become mandatory to improve vaccine coverage in the livestock population. It is suggested to sequence the virus more often to monitor changes in the circulating FMDV strains in order to effectively select vaccines strains. Furthermore, vaccination should be performed before the start of the rainy season to ensure that animals develop immunity before the high‐risk period. As vaccination is performed by farmers in some areas, clear instructions should be given to ensure proper cold chain storage and vaccine administration. The potency and efficacy of the vaccine in the field should be monitored to ensure herd immunity. FMD awareness by farmers should be promoted to improve passive surveillance and uptake of vaccination. Concurrently, active surveillance of FMD should be planned for early detection and outbreak response.

## 5 CONCLUSIONS

FMD hinders livestock production in Thailand as outbreaks have been occurring every year, despite routine vaccination and control programmes. The occurrence of FMD outbreaks was affected by season and spatially clustered in certain areas with underlying risk factors. Risk factors associated with FMD outbreak included the population size of certain livestock species, the size of the forest area, international border, a history of an FMD outbreak in neighbouring subdistricts, the proximity to FMD outbreaks in a previous month and the presence of an animal market. The insight into the pattern and risk factors of FMD outbreak can help to manage resources to the high‐risk areas and periods in Thailand.

The programming codes for models are available at https://github.com/AnnThanicha/FMD‐spatial‐temporal‐Thailand.

## AUTHOR CONTRIBUTIONS

Thanicha Chanchaidechachai and Bart H. P. van den Borne: design study, analyse data and write original draft. Helmut Saatkamp, Henk Hogeveen, Chaidate Inchaisri and Mart de Jong: design study, review and edit manuscript. Sith Premashthira, Noppawan Buamitoup and Rotchana Prakotcheo: collect the data, review and edit manuscript. All authors contributed to the article and approved the submitted version.

## CONFLICT OF INTEREST

The authors declare that there is no conflict of interest.

## ETHICS STATEMENT

The authors have declared that an Ethical Statement is not applicable for the current study.

## Data Availability

The datasets generated for this study have been provided by the authority of the Department of Livestock Development, Ministry of Agriculture and Cooperatives, Thailand. Requests to access these datasets should be directed to this organization.

## References

[tbed14754-bib-1001] Aiewsakun, P. , Pamornchainavakul, N. , & Inchaisri, C. (2020). Early origin and global colonisation of foot‐and‐mouth disease virus. Scientific Reports, 10, 15268. 10.1038/s41598-020-72246-6 32943727 PMC7498456

[tbed14754-bib-0001] Allepuz, A. , Stevenson, M. , Kivaria, F. , Berkvens, D. , Casal, J. , & Picado, A. (2015). Risk factors for foot‐and‐mouth disease in Tanzania, 2001–2006. Transboundary and Emerging Diseases, 62(2), 127–136. 10.1111/tbed.12087 23621861

[tbed14754-bib-0002] Angkuraseranee, T. , Somboonsuk, B. , Sukhabot, S. , & Nimsai, S. (2019). Market opportunities for Thai beef cattle exports to Yunnan province, China. International Journal of Agricultural Technology, 15(6), 807–822. Retrieved from http://www.ijat‐aatsea.com/pdf/v15_n6_2019_November/1_IJAT_15(6)_2019_Angkuraseranee, T..pdf

[tbed14754-bib-0003] Arjkumpa, O. , Sansamur, C. , Sutthipankul, P. , Inchaisri, C. , Na Lampang, K. , Charoenpanyanet, A. , & Punyapornwithaya, V. (2020). Spatiotemporal analyses of foot and mouth disease outbreaks in cattle farms in Chiang Mai and Lamphun, Thailand. BMC Veterinary Research, 16(1), 1–13. 10.1186/s12917-020-02392-6 32487166 PMC7268379

[tbed14754-bib-0004] Arjkumpa, O. , Yano, T. , Prakotcheo, R. , Sansamur, C. , & Punyapornwithaya, V. (2020). Epidemiology and national surveillance system for foot and mouth disease in cattle in Thailand during 2008–2019. Veterinary Sciences, 7(3), 99. 10.3390/vetsci7030099 32722145 PMC7558286

[tbed14754-bib-0005] Ayebazibwe, C. , Tjørnehøj, K. , Mwiine, F. N. , Muwanika, V. B. , Ademun Okurut, A. R. , Siegismund, H. R. , & Alexandersen, S. (2010). Patterns, risk factors and characteristics of reported and perceived foot‐and‐mouth disease (FMD) in Uganda. Tropical Animal Health and Production, 42(7), 1547–1559. 10.1007/s11250-010-9605-3 20526861

[tbed14754-bib-0006] Bivand, R. S. , Pebesma, E. , & Gómez‐Rubio, V. (2013). Applied spatial data analysis with R. Springer New York. 10.1007/978-1-4614-7618-4

[tbed14754-bib-0007] Blacksell, S. D. , Siengsanan‐Lamont, J. , Kamolsiripichaiporn, S. , Gleeson, L. J. , & Windsor, P. A. (2019). A history of FMD research and control programmes in Southeast Asia: Lessons from the past informing the future. Epidemiology and Infection, 147, e171. 10.1017/S0950268819000578 31063108 PMC6499730

[tbed14754-bib-0008] Bravo de Rueda, C. , de Jong, M. C. , Eblé, P. L. , & Dekker, A. (2015). Quantification of transmission of foot‐and‐mouth disease virus caused by an environment contaminated with secretions and excretions from infected calves. Veterinary Research, 46(1), 43. 10.1186/s13567-015-0156-5 25928658 PMC4404111

[tbed14754-bib-0009] Bunmee, T. , Chaiwang, N. , Kaewkot, C. , & Jaturasitha, S. (2018). Current situation and future prospects for beef production in Thailand – A review. Asian‐Australasian Journal of Animal Sciences, 31(7), 968–975. 10.5713/ajas.18.0201 29879818 PMC6039331

[tbed14754-bib-0010] Clayton, D. , & Kaldor, J. (1987). Empirical Bayes estimates of age‐standardized relative risks for use in disease mapping. Biometrics, 43(3), 671–81. Retrieved from http://www.jstor.com/stable/2532003 3663823

[tbed14754-bib-0011] Cleveland, R. B. , Cleveland, W. S. , McRae, J. E. , & Terpenning, I. (1990). STL: A seasonal‐trend decomposition procedure based on loess. Journal of Official Statistics, 6(1), 3–73. Retrieved from https://search.proquest.com/docview/1266805989?pq‐origsite=gscholar&fromopenview=true

[tbed14754-bib-0012] Cui, J. , & Qian, G. (2007). Selection of working correlation structure and best model in GEE analyses of longitudinal data. Communications in Statistics: Simulation and Computation, 36(5), 987–996. 10.1080/03610910701539617

[tbed14754-bib-0013] Davies, G. (2002). Foot and mouth disease. Research in Veterinary Science, 73(3), 195–199. 10.1016/S0034-5288(02)00105-4 12443674

[tbed14754-bib-0014] Delgado, C. L. , Narrod, C. A. , Tiongco, M. , Sant'Ana de Camargo Barros, G. , Catelo, M. A. O. , Costales, A. , Mehta, R. , Naranong, V. , Poapongsakorn, N. , Sharma, V. P. , & de Zen, S. (2008). Determinants and implications of the growing scale of livestock farms in four fast‐growing developing countries. International Food Policy Research Institute. 10.2499/9780896291669RR157

[tbed14754-bib-0015] Department of Livestock Development (DLD) . (2019). DLD website for predictions. DLD. Retrieved 04/10/2020 from http://predict.dld.go.th/

[tbed14754-bib-0016] Department of Livestock Development . (2020). ข้อมูลจำนวนปศุสัตว์ในประเทศไทย ปี 2563 . Retrieved 01/12/2021 from http://docimage.dld.go.th/FILEROOM/CABDLD_BOOKSHELF2/DRAWER26/GENERAL/DATA0000/00000082.PDF

[tbed14754-bib-0017] Estrada, C. , Perez, A. M. , & Thurmond, M. C. (2008). Herd reproduction ratio and time‐space analysis of a foot‐and‐mouth disease epidemic in Peru in 2004. Transboundary and Emerging Diseases, 55(7), 284–292. 10.1111/j.1865-1682.2008.01023.x 18631231

[tbed14754-bib-0018] Gómez‐Rubio, V. , Ferrándiz‐Ferragud, J. , & López‐Quílez, A. (2005). Detecting clusters of disease with R. Journal of Geographical Systems, 7(2), 189–206. 10.1007/s10109-005-0156-5

[tbed14754-bib-0019] Guerrini, L. , Pfukenyi, D. M. , Etter, E. , Bouyer, J. , Njagu, C. , Ndhlovu, F. , Bourgarel, M. , de Garine‐Wichatitsky, M. , Foggin, C. , Grosbois, V. , & Caron, A. (2019). Spatial and seasonal patterns of FMD primary outbreaks in cattle in Zimbabwe between 1931 and 2016. Veterinary Research, 50(1), 1–12. 10.1186/s13567-019-0690-7 31551078 PMC6760110

[tbed14754-bib-0020] Hamoonga, R. , Stevenson, M. A. , Allepuz, A. , Carpenter, T. E. , & Sinkala, Y. (2014). Risk factors for foot‐and‐mouth disease in Zambia, 1981–2012. Preventive Veterinary Medicine, 114(1), 64–71. 10.1016/j.prevetmed.2014.01.014 24486093

[tbed14754-bib-0021] Han, J. , Zhu, L. , Kulldorff, M. , Hostovich, S. , Stinchcomb, D. G. , Tatalovich, Z. , Lewis, D. R. , & Feuer, E. J. (2016). Using Gini coefficient to determining optimal cluster reporting sizes for spatial scan statistics. International Journal of Health Geographics, 15(1), 1–11. 10.1186/s12942-016-0056-6 27488416 PMC4971627

[tbed14754-bib-0022] Hyndman, R. J. , & Athanasopoulus, G. (2018). Forecasting: Principles and practice. OTexts. Retrieved 02/16/2021 from https://otexts.com/fpp2/

[tbed14754-bib-0023] Jemberu, W. T. , Mourits, M. C. M. , Sahle, M. , Siraw, B. , Vernooij, J. C. M. , & Hogeveen, H. (2016). Epidemiology of foot and mouth disease in Ethiopia: A retrospective analysis of district level outbreaks, 2007–2012. Transboundary and Emerging Diseases, 63(6), e246–e259. 10.1111/tbed.12338 25704390

[tbed14754-bib-0024] Knight‐Jones, T. J. D. D. , Bulut, A. N. , Gubbins, S. , Stärk, K. D. C. C. , Pfeiffer, D. U. , Sumption, K. J. , & Paton, D. J. (2015). Randomised field trial to evaluate serological response after foot‐and‐mouth disease vaccination in Turkey. Vaccine, 33(6), 805–811. 10.1016/j.vaccine.2014.12.010 25528523 PMC4334422

[tbed14754-bib-0025] Knowles, N. J. , He, J. , Shang, Y. , Wadsworth, J. , Valdazo‐González, B. , Onosato, H. , Fukai, K. , Morioka, K. , Yoshida, K. , Cho, I. S. , Kim, S. M. , Park, J. H. , Lee, K. N. , Luk, G. , Borisov, V. , Scherbakov, A. , Timina, A. , Bold, D. , Nguyen, T. , …, King, D. P. (2012). Southeast Asian foot‐and‐mouth disease viruses in Eastern Asia. Emerging Infectious Diseases, 18(3), 499–501. 10.3201/eid1803.110908 22377196 PMC3309575

[tbed14754-bib-0026] Kulldorff, M. (2018). SaTScanTM *user guide for ver*sion 9.6. Retrieved 10/12/2020 from https://www.satscan.org/cgi‐bin/satscan/register.pl/SaTScan_Users_Guide.pdf?todo=process_userguide_download

[tbed14754-bib-0027] Kulldorff, M. (2020). SaTScan. Harvard Medical School and Harvard Pilgrim Health Care. Retrieved from http://www.satscan.org

[tbed14754-bib-0028] Madin, B. (2011). An evaluation of foot‐and‐mouth disease outbreak reporting in mainland South‐East Asia from 2000 to 2010. Preventive Veterinary Medicine, 102(3), 230–241. 10.1016/j.prevetmed.2011.07.010 21889809

[tbed14754-bib-0029] Mahapatra, M. , & Parida, S. (2018). Foot and mouth disease vaccine strain selection: Current approaches and future perspectives. Expert Review of Vaccines, 17(7), 577–591. 10.1080/14760584.2018.1492378 29950121

[tbed14754-bib-0030] Megersa, B. , Beyene, B. , Abunna, F. , Regassa, A. , Amenu, K. , & Rufael, T. (2009). Risk factors for foot and mouth disease seroprevalence in indigenous cattle in Southern Ethiopia: The effect of production system. Tropical Animal Health and Production, 41(6), 891–898. 10.1007/s11250-008-9276-5 19052894

[tbed14754-bib-0031] Mielke, S. R. , & Garabed, R. (2020). Environmental persistence of foot‐and‐mouth disease virus applied to endemic regions. Transboundary and Emerging Diseases, 67(2), 543–554. 10.1111/tbed.13383 31595659

[tbed14754-bib-0032] Nyaguthii, D. M. , Armson, B. , Kitala, P. M. , Sanz‐Bernardo, B. , Di Nardo, A. , & Lyons, N. A. (2019). Knowledge and risk factors for foot‐and‐mouth disease among small‐scale dairy farmers in an endemic setting. Veterinary Research, 50(1), 1–12. 10.1186/s13567-019-0652-0 31088554 PMC6518695

[tbed14754-bib-0033] Perry, B. D. , Kalpravidh, W. , Coleman, P. G. , Horst, H. S. , McDermott, J. J. , Randolph, T. F. , & Gleeson, L. J. (1999). The economic impact of foot and mouth disease and its control in South‐East Asia: A preliminary assessment with special reference to Thailand. OIE Revue Scientifique et Technique, 18(2), 478–497. 10.20506/rst.18.2.1163 10472680

[tbed14754-bib-0034] Picado, A. , Speybroeck, N. , Kivaria, F. , Mosha, R. M. , Sumaye, R. D. , Casal, J. , & Berkvens, D. (2011). Foot‐and‐mouth disease in Tanzania from 2001 to 2006. Transboundary and Emerging Diseases, 58(1), 44–52. 10.1111/j.1865-1682.2010.01180.x 21078082

[tbed14754-bib-0035] Pomeroy, L. W. , Bjørnstad, O. N. , Kim, H. , Jumbo, S. D. , Abdoulkadiri, S. , & Garabed, R. (2015). Serotype‐specific transmission and waning immunity of endemic foot‐and‐mouth disease virus in Cameroon. PLoS One, 10(9), e0136642. 10.1371/journal.pone.0136642 26327324 PMC4556668

[tbed14754-bib-0036] QGIS Development Team . (2020). QGIS geographic information system. Open Source Geospatial Foundation Project. Retrieved from http://qgis.osgeo.org

[tbed14754-bib-0037] R Core Team . (2022). R: A language and environment for statistical computing. R Foundation for Statistical Computing. Retrieved from https://www.r‐project.org/

[tbed14754-bib-0038] Sangrat, W. , Thanapongtharm, W. , & Poolkhet, C. (2020). Identification of risk areas for foot and mouth disease in Thailand using a geographic information system‐based multi‐criteria decision analysis. Preventive Veterinary Medicine, 185(October), 105183. 10.1016/j.prevetmed.2020.105183 33153767

[tbed14754-bib-0039] Sansamur, C. , Arjkumpa, O. , Charoenpanyanet, A. , & Punyapornwithaya, V. (2020). Determination of risk factors associated with foot and mouth disease outbreaks in dairy farms in Chiang Mai Province, Northern Thailand. Animals, 10(3), 512. 10.3390/ani10030512 32204373 PMC7143784

[tbed14754-bib-0040] Siengsanan‐Lamont, J. , & Blacksell, S. D. (2021). Surveillance for One Health and high consequence veterinary pathogens (Brucellosis, Coxiellosis and Foot and Mouth Disease) in Southeast Asia: Lao PDR and Cambodia in focus and the importance of international partnerships. Microbiology Australia, 42(4), 156. 10.1071/MA21045

[tbed14754-bib-0041] Smith, P. , Luthi, N. B. , Li, H. , Kyaw, N. O. , Phonvisay, A. , Premashthira, S. , Abila, R. , Widders, P. , Kukreja, K. , & Miller, C. (2015). Movement pathways and market chains of large ruminants in the Greater Mekong Sub‐region. Retrieved 10/12/2020 from https://rr‐asia.oie.int/wp‐content/uploads/2019/10/livestock_movement_pathways_and_markets_in_the_gms__final_.pdf

[tbed14754-bib-0042] Souriya, V. , Piamsomboon, P. , Ajariyakhajorn, K. , Damrongwatanapokin, T. , & Inchaisri, C. (2020). Risk factors of foot and mouth disease in an endemic area on low vaccination rate in Xayaboury province of Lao People's Democratic Republic (Lao PDR). Tropical Animal Health and Production, 52(3), 1103–1114. 10.1007/s11250-019-02113-8 31729631

[tbed14754-bib-0043] Stolwijk, A. M. , Straatman, H. , & Zielhuis, G. A. (1999). Studying seasonality by using sine and cosine functions in regression analysis. Journal of Epidemiology and Community Health, 53(4), 235–238. 10.1136/jech.53.4.235 10396550 PMC1756865

[tbed14754-bib-0044] Thai Meteorological Department . (2015). The climate of Thailand. Thai Meteorological Department. Retrieved 02/16/2021 from https://www.tmd.go.th/en/thailand.php

[tbed14754-bib-0045] van Andel, M. , Zaari, S. , Bernard, P. , McFadden, A. , Dacre, I. , Bingham, P. , Heuer, C. , Binney, B. , Buckle, K. , Abila, R. , Win, H. H. , Lwin, K. O. , & Gates, M. C. (2020). Evaluating the utility of national‐scale data to estimate the local risk of foot‐and‐mouth disease in endemic regions. Transboundary and Emerging Diseases, 67(1), 108–120. 10.1111/tbed.13329 31408585

[tbed14754-bib-0046] Waller, L. A. , & Gotway, C. A. (2004). Applied spatial statistics for public health data. John Wiley & Sons, Inc. 10.1002/0471662682

[tbed14754-bib-0047] Wataradee, S. , Boonserm, T. , Srangaprakon, C. , Ajariyakhajorn, K. , & Inchaisri, C. (2021). Use of an automatic needle‐free injection device for foot‐and‐mouth disease vaccination in dairy heifers. Veterinární Medicína, 66(3), 87–93. 10.17221/4/2020-VETMED PMC11975267 40201432

[tbed14754-bib-1047] World Organisation for Animal Health (WAOH) . (2017). SEACFMD Bulletin: Foot and Mouth Disease Situation January to December 2016 [Online] Available at https://rr-asia.oie.int/wp-content/uploads/2019/10/2016_seacfmd_bulletin.pdf (accessed June 26, 2021). Bangkok.

[tbed14754-bib-0048] Yano, T. , Premashthira, S. , Dejyong, T. , Tangtrongsup, S. , & Salman, M. D. (2018). The effectiveness of a foot and mouth disease outbreak control programme in Thailand 2008–2015: Case studies and lessons learned. Veterinary Sciences, 5(4), 1–13. 10.3390/vetsci5040101 30563300 PMC6313864

